# Sustainable production of intermetallic nanocomposites from aluminum and iron scarp, with excellent tribo-mechanical and thermal properties for industrial applications

**DOI:** 10.1038/s41598-025-17118-7

**Published:** 2025-09-12

**Authors:** Rasha A. Youness, Mohammed A. Taha

**Affiliations:** 1 Spectroscopy Department, National Research Centre, El Buhouth St, Dokki, Giza, 12622 Egypt; 2 Solid State Physics Department, National Research Centre, El Buhouth St, Dokki, Giza, 12622 Egypt; 3 Pharos University in Alexandria, Canal Mahmoudiah Street, Smouha, Egypt

**Keywords:** Intermetallic alloy, Nanocomposites, Waste materials, Mechanical properties, Wear resistance, Powder metallurgy, Materials science, Physics

## Abstract

The goal of this research is to convert iron (Fe) and aluminum (Al) waste from metal workshops, as well as silicon (Si), into innovative intermetallic nanocomposites with different characteristics that are reinforced with various quantities of hybrid fly ash and vanadium carbide (VC) for industrial usage. The microstructure, physical integrity, tribo-mechanical performance, and thermal behavior of the resultant sintered materials were all carefully examined. FeSi, FeAl, Fe₃Si, and Fe₃Al₂Si₃ were among the intermetallic phases that were formed, according to post-milling XRD examination. The bulk density of the intermetallic alloy steadily dropped as the amount of hybrid ceramic reinforcements rose, although the apparent porosity in the sintered microstructure increased. Notably, as compared to the unreinforced intermetallic alloy, the microhardness increased by 8.11%, 23.62%, 47.10%, and 84.26% in quick succession when these reinforcements were added. In comparison to the intermetallic alloy, the sample containing 16 vol% of hybrid reinforcements (FV8) achieved a Young’s modulus of 84.1% and a compressive strength of 43.2% after the addition of reinforcements. The CTE value of the intermetallic alloy was 11.88 × 10^−6^/ ⁰C, whereas the nanocomposite samples FV1, FV2, FV4 and FV8 have values of 11.28 × 10^−6^, 10.61 × 10^−6^, 9.14 × 10^−6^, and 7.10 × 10^−6^/ ⁰C, respectively, which can be attributed to vanadium and silica, which have lower CTE values than the matrix. Moreover, the previous results are associated with improved tribological properties of the prepared nanocomposites, as their wear rate decreased by 4.6%, 10.9%, 22.8%, and 43.2% compared to the intermetallic alloy. The average fraction coefficient decreased by 5.3%, 11.9%, 22.4%, and 39.5% for the same samples. Based on the results, recycled materials can be used in industrial applications, reinforcing the importance of recycling metal waste.

## Introduction

In light of growing environmental challenges and the urgent need for more resource-efficient technologies, the shift toward sustainable materials has become essential across various industrial sectors. While raw materials remain fundamental to supporting industrial development, their extraction and processing are associated with high energy consumption and significant environmental harm. Consequently, increasing attention has been directed toward developing alternative solutions based on sustainability principles. One of the most prominent modern approaches in this context is the development of sustainable nanocomposite materials, which are produced using recycled metals, industrial waste, and renewable bio-based resources^1–4^. Recent studies^5–8^ have introduced innovative models for synthesizing nanocomposites through green chemistry techniques, the reuse of agricultural and industrial waste, and energy-efficient processing methods. These nanocomposites balance environmental responsibility and high functional performance, making them suitable for various applications such as packaging, biomedical purposes, and structural components.

Metal recycling is considered one of the key applications that support this sustainable direction, not only as a source of raw materials for nanocomposite production but also as an effective means of reducing environmental impact. Research has shown that re-melting scrap metals consumes 60–95% less energy than primary smelting, significantly reducing greenhouse gas emissions and decreasing reliance on mining, often in environmentally sensitive areas. Furthermore, recycling helps mitigate habitat destruction, soil erosion, and acid mine drainage, reducing solid waste and preventing heavy metal leakage into groundwater. In this way, recycling contributes to closing material loops and advancing climate action goals by creating sustainable, low-impact industrial systems^9^.

Due to their outstanding wear resistance, high strength, good oxidation resistance at high temperatures, excellent soft magnetic properties, and low cost, intermetallic compounds based on Fe, especially iron aluminides (Fe-Al) and iron silicides (Fe-Si), are thought to be up-and-coming materials for industrial applications where they can be used as a substitute for stainless steels and/or nickel-based superalloys^10–14^. Moreover, the addition of Si to the Fe-Al alloy has a positive effect as it is considered an alloying element and increases the electrical and thermal conductivity in addition to improving the mechanical properties and stability of the microstructure against grain coarsening due to the formation of silicon precipitates^10,15^. From this point of view, the need to enhance the strength, CTE, and fraction coefficient of Fe-Al-Si intermetallic alloy has become increasingly demanded due to the need for it in various industrial applications such as for resistance heating elements, furnace components, parts of valve systems, exhaust pipes, heat exchangers in turbine systems, etc. Although the pleasing qualities of Fe-Al-Si intermetallic alloys are becoming increasingly clear-cut and in demand, conventional processing is insufficient to prepare them reliably, efficiently, and inexpensively^13,16–18^.

VC is a commercially important ceramic because of its extreme strength, corrosion resistance, and high thermal stability, and it is an excellent reinforcement for metal refinement^19–21^. Low-cost, low-density fly ash particulate reinforcements can be used as alternatives to relatively more expensive reinforcements to reduce costs. Fly ash is a byproduct of the industrial process of burning coal in electric power plants. The main components of fly ash are oxides and mixed metal oxides of Si, Al, and Fe, and thus, it is characterized by high strength, high wear resistance, and a low CTE value^22,23^. A promising method for preparing intermetallic alloys reinforced by hybrid ceramic particles that may exhibit superior mechanical properties and wear resistance is powder metallurgy (PM). However, interface bonding between hybrid ceramic particles and intermetallic matrix is always an issue in nanocomposites prepared through PM^24,25^. Generally, utilizing the PM method is an effective way to prepare nanocomposites that are difficult to obtain by conventional methods. Moreover, this method is cost-effective for manufacturing^26–28^. Notably, this method allows the preparation of the nanocomposites based on intermetallics, with the formation of the final stage starting from the mixture of the starting elements. This is usually achieved using high-energy ball milling^10,29^. It is worth noting that obtaining nanocomposites has become expensive due to the high cost of raw materials.

Based on the concepts above, the novelty of this work is recycling waste metals peels (i.e., Fe and Al) and ceramics (fly ash) to produce a nanocomposite based on Fe20Al20Si intermetallic alloy reinforced with various volume fractions of hybrid VC and fly ash using the powder metallurgy method. In addition to researching how these reinforcements affect the microstructure of the powder and sintered formations, the intermetallic alloy’s mechanical and physical characteristics, wear resistance, and thermal expansion coefficient are also studied.

Based on the concepts above, the novelty of this work is recycling waste metals peels (i.e., Fe and Al) and ceramics (fly ash) to produce a nanocomposite based on Fe20Al20Si intermetallic alloy reinforced with various volume fractions of hybrid VC and fly ash using the PM method. In addition to researching how these reinforcements affect the microstructure of the powder and sintered formations, the intermetallic alloy’s mechanical and physical characteristics, wear resistance, and thermal expansion coefficient are also considered. Based on these investigations, this study contributes to overcoming global sustainability challenges by promoting resource efficiency, reducing industrial waste, and supporting the development of eco-friendly, high-performance materials from recycled inputs.

## Materials and methods

### Powders preparation

**Fig. 1:**
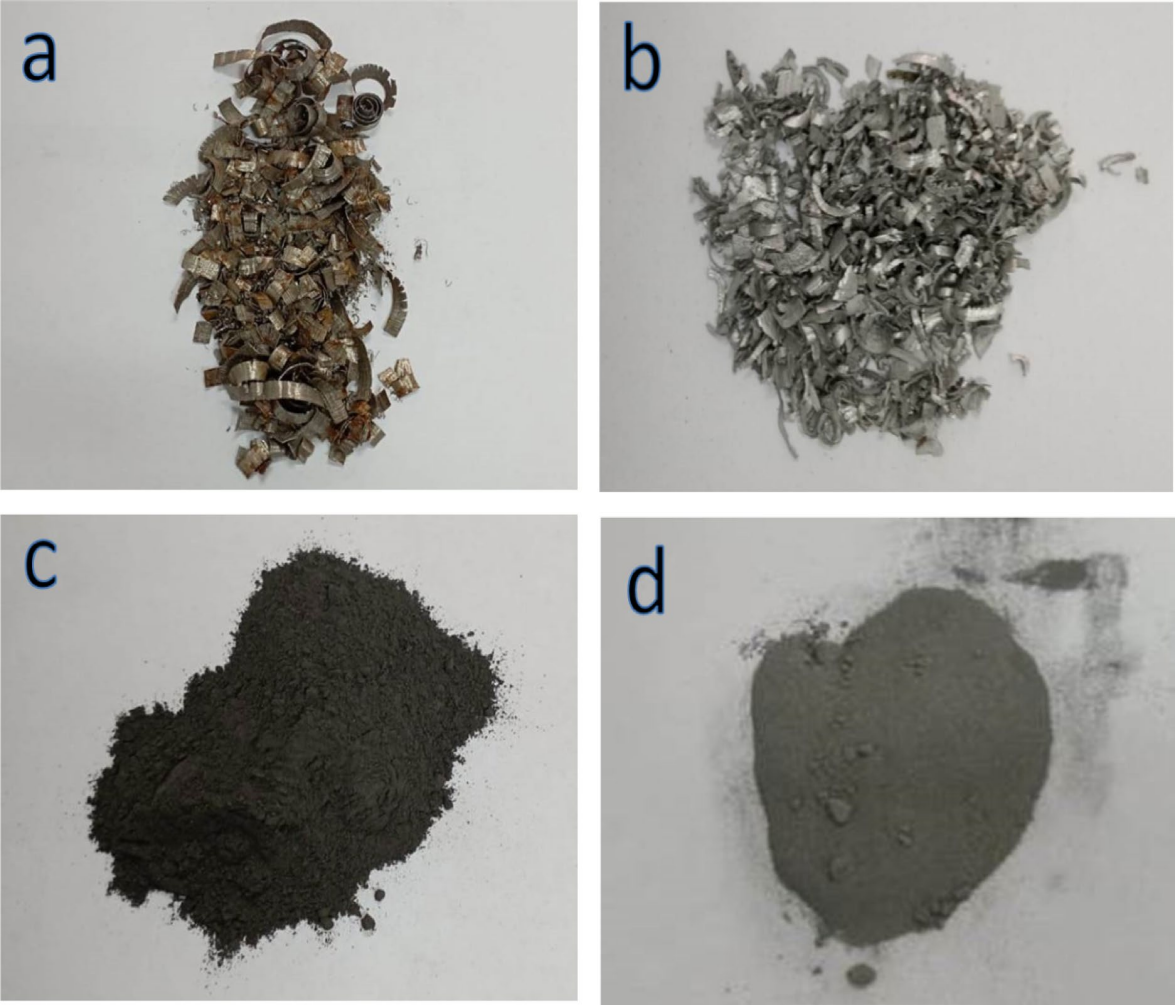
Photos of Fe and Al waste generated in lathe workshops (**a**,** b**) and their photos after grinding for three hours (**c**,** d**).

**Fig. 2:**
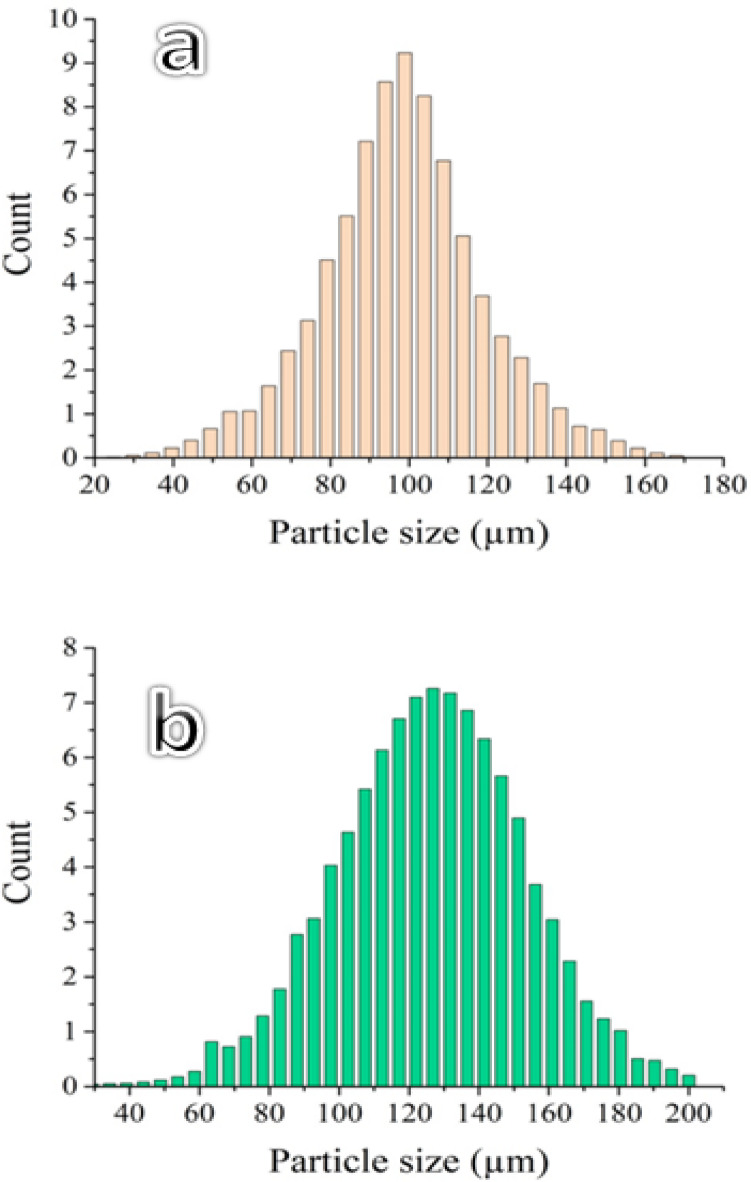
Average particle size of Fe and Al wastes from grinding was obtained from a laser diffraction particle size analyzer.

The Fe and Al waste from lathe workshops was initially broken up into small fragments, several millimeters in size, using a ball mill for three hours (Fig. 1), with an average particle size of about 95 and 130 μm, respectively, using a diffraction particle size analyzer (Fig. 2). The particle size of Si and VC is 50 μm and 40.5 nm, respectively, and fly ash is 32.4 nm to 81.2 nm. The particle sizes of VC are 50 and 40.5 nm, respectively, and fly ash is 32.4 nm to 81.2 nm. Firstly, the base matrix of this work was FeAl20Si20 (wt%) alloy mixed for 20 h with a ball-to-powder ratio equal to 5:1 and a speed of 150 rpm. Secondly, varied contents of the hybrid fly ash and VC were introduced to the FeAl20Si20 alloy matrix. The composition of Fe, Al, and fly ash waste powder (wt%) is tabulated in Tables 1 and 2. The batch compositions designed for nanocomposites, with their abbreviations, are tabulated in Table 3. Each sample powder was milled for 20 h at 500 rpm with a ratio of balls to powder = 20:1. The powder was compacted with a hydraulic machine with a pressure of 440 bar and then sintered in an argon atmosphere for one hour at 1100 °C.

**Table 1:** Composition of Fe and al waste powder (wt%).

	Fe waste								
Element	Fe	Mg	Al	Mn	Si	C	P	others	
wt%	99.84	0.02	0.04	0.03	0.01	0.01	0.02	0.03	
	***Al waste***								
Element	Al	Si	Mg	Cu	Zn	Fe	Ti	Nb	others
wt%	99.81	0.04	0.02	0.02	0.01	0.04	0.01	0.01	0.04

**Table 2:** Composition of fly Ash powder (wt%).

Element	SiO_2_	Al_2_O_3_	Fe_2_O_3_	CaO	MgO	K_2_O	TiO_2_	Others
wt%	62.81	18.27	6.20	5.01	2.61	3.44	1.55	0.11

**Table 3:** Batch design of the prepared samples.

Sample code	Fe20Al20Si	VC	Fly ash
FV0	100	0	0
FV1	98	1	1
FV2	96	2	2
FV4	92	4	4
FV8	84	8	8

### Characteristics of raw and milled powders

X-ray diffraction (Philips PW 1373) technique was utilized to investigate the phase composition of the stated and the prepared powders. The crystal size (C), lattice strain (ε), and dislocation density (δ) were calculated from the X-ray line broadening (B) for the principle (*h k l*) planes using the equations according to a recent paper^30,31^.

1 $$\:\text{C}=\:\frac{0.9{\uplambda\:}}{\text{B}\text{c}\text{o}\text{s}{\uptheta\:}}$$

2 $$\:{\upepsilon\:}=\:\frac{\text{B}}{4\:\text{t}\text{a}\text{n}{\uptheta\:}}$$

3 $${\updelta\:}=\:\frac{1}{\text{C}}$$

where the wavelength, i.e., λ ꞊ 0.1540591 nm (Cu-Ni radiation) and θ is the diffraction angle in rad.

High-resolution transmission electron microscopy coupled with selected area electron diffraction (HRTEM-SAED; HRJEOL JEM-2100) was used to investigate the particle size.

### Microstructure of sintered nanocomposite samples

Field emission scanning electron microscopy (FESEM) coupled with energy dispersive X-ray analysis **(**EDX) (type Quanta FEG250) was also used to examine the microstructure of sintered intermetallic alloy and its nanocomposites.

### Measurement of the different properties of sintered nanocomposite samples

#### Physical properties

The Archimedes method (ASTM: B962-13) examined sintered samples’ relative density and apparent porosity.

#### Thermal analysis

Thermal expansion measurements of the sintered intermetallic alloy and its nanocomposites were analyzed from 30 to 1000 °C in air, and the estimated CTE values were evaluated.

#### Mechanical properties

The Vickers tester was utilized to determine the prepared specimens’ microhardness (Hv) value following ASTM B933-09, using Eq. 4^32,33^.

4 $$\:\text{H}\text{v}=1.854\:\times\:\:\frac{\text{P}}{{\text{d}}^{2}}$$

where P is the load and d is the diagonal of indentation.

The compressive strength of the prepared specimens has been evaluated according to ASTM E9–19. The prepared specimens’ longitudinal (V_L_) and shear (V_S_) ultrasonic velocities were also measured using the pulse-echo technique. The values of *λ* and *µ*were calculated by equations^34,35^.

5 $$\:{\uplambda\:}=\text{B}.\text{D}\:\times\:\:({V}_{L}^{2}-2{\text{V}}_{S}^{2})$$

6 $$\:{\upmu\:}\:=\text{B}.\text{D}\:\times\:\:{V}_{S}^{2}\:$$

The values of the elastic moduli, longitudinal modulus (*L*), shear modulus (*G*), Young’s modulus (*E*), and bulk modulus (*B*) were calculated from the following equations^36–38^:

7 $$\:L=\lambda\:+2\mu\:$$

8 $$\:G=\mu\:$$

9 $$\:E=\mu\:\frac{3\lambda\:+2\mu\:}{\lambda\:+\mu\:}$$

10 $$\:B=\lambda\:+\frac{2}{3}\mu\:$$

#### Wear test

**Fig. 3:**
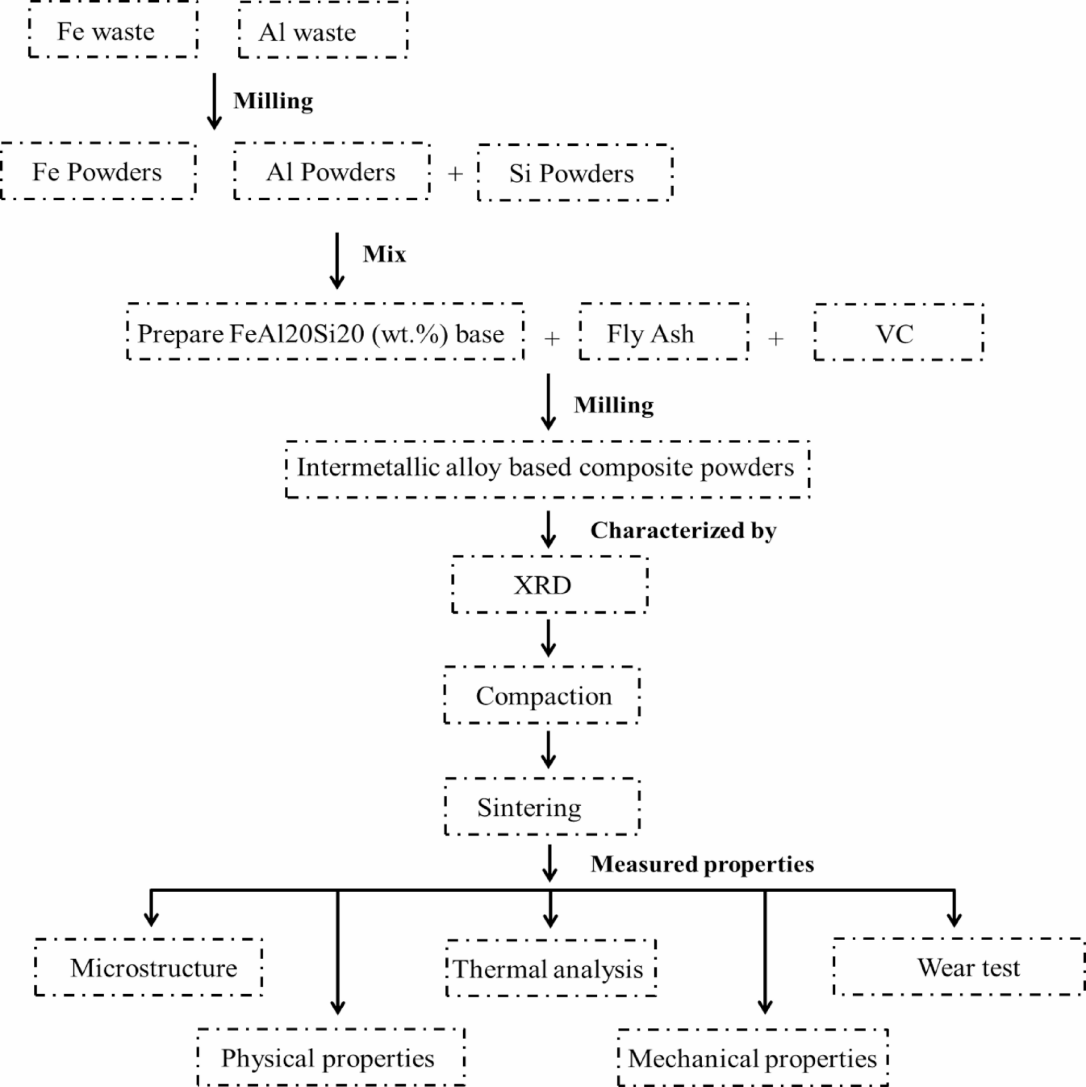
The schematic diagram illustrates the sample preparation procedures.

The wear test was conducted using a pin-on-disk tester machine under an applied load of 20 N and a speed of 0.8 m/s for 10 min. The coefficient of the fraction was measured, and the wear rate was calculated using the article^39^. It is worth noting that the preparation method, the techniques used in characterization, and the various properties measured in this research for the prepared samples are summarized in Fig. 3.

## Results and discussion

### Investigation of raw and prepared powders

**Fig. 4:**
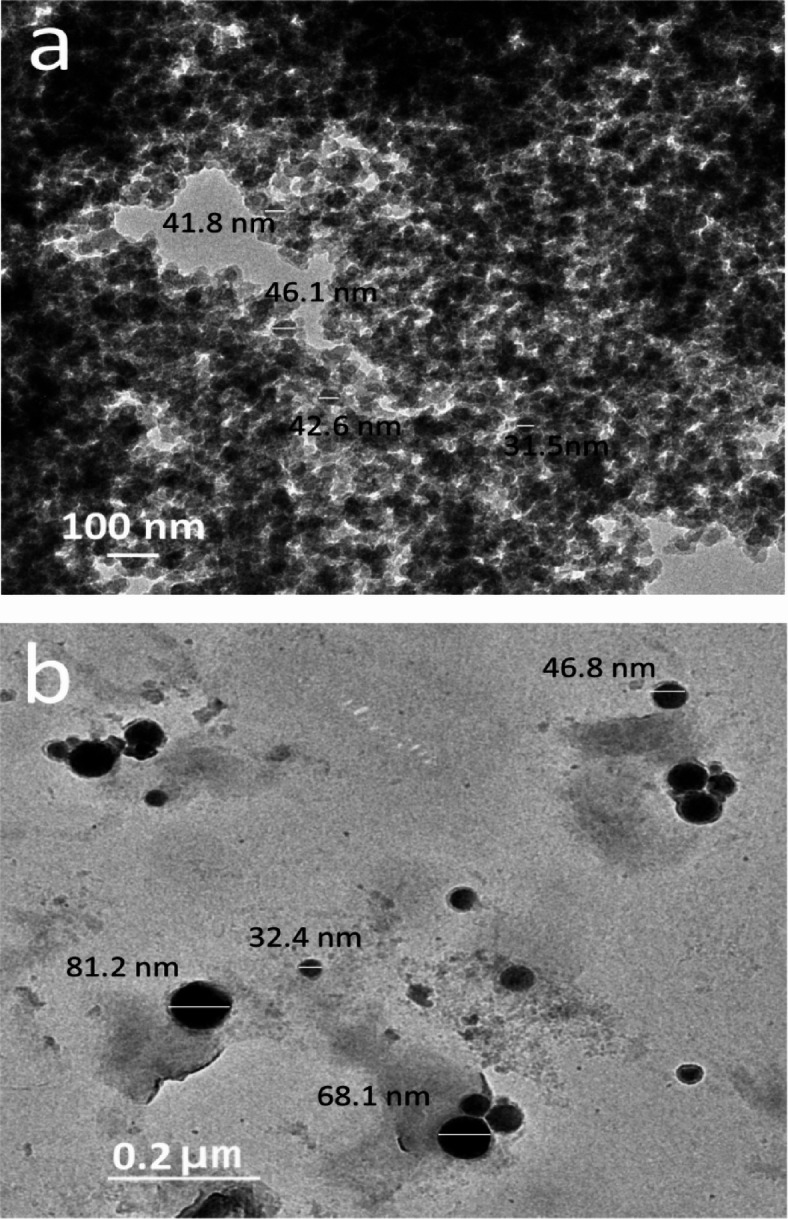
The HRTEM images of **a**) VC powder, **b**) fly ash reinforcement powder.

Figure 4(a, b) shows TEM images of VC and fly ash ceramics. The reinforcement ceramics appear in particles; the average particle size of VC is 40.5 nm, while the particle size of fly ash is 32.4 nm to 81.2 nm.

**Fig. 5:**
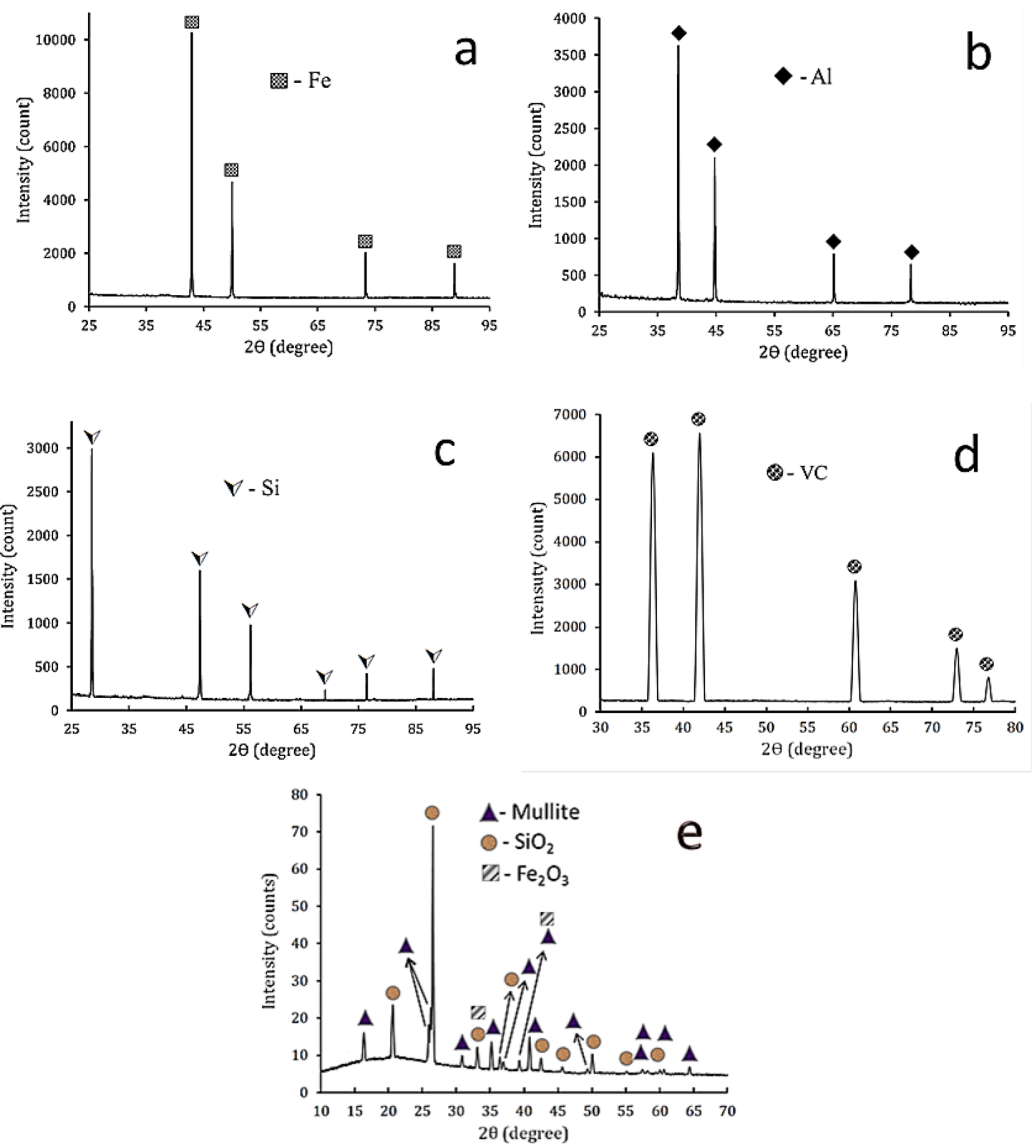
The XRD patterns of the raw materials, i.e., **a**) Fe, **b**) Al, **c**) Si, **d**) VC, and **e**) fly ash powders.

**Fig. 6:**
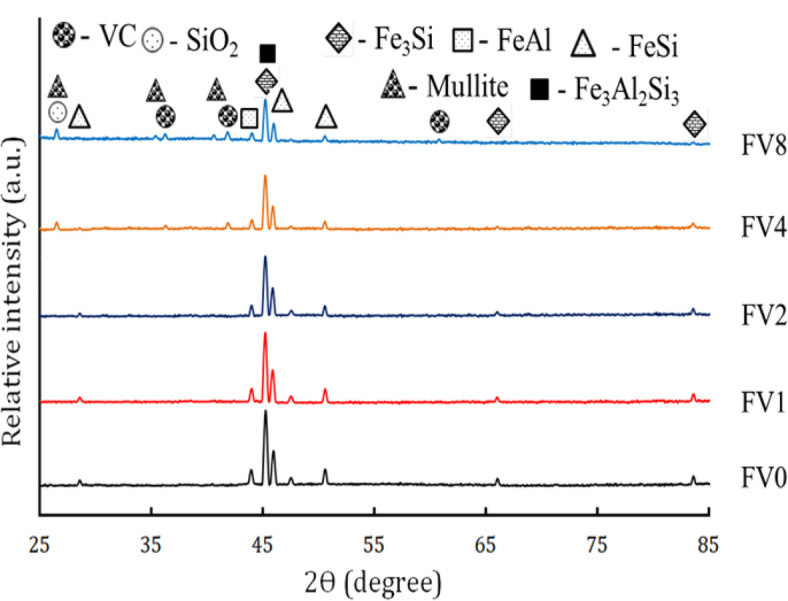
XRD patterns of the FeAl20Si20 alloy and its hybrid nanocomposites with varying fly ash and VC contents after 20 h of milling.

During grinding, waste metal peels undergo significant changes such as repeated deformation, cold joining, and fracture by ball impact. It is noteworthy that the notable rise in average particle size can be attributed to the superiority of welding development. Nonetheless, the average particle size is declining due to the dominance of the fracture process. Figure 5(a-e) shows the XRD of the starting powder, including Fe, Al, Si, VC, and fly ash. The figure shows peaks corresponding to Fe, Al, Si, and VC powders according to (ICCD file cards: 89-4185, 89-4037, 89-5012, and 74-1220), respectively. The fly ash chart showed that SiO_2_ mullite and Fe_2_O_3_, along with amorphous silica, were the fly ash’s two main crystalline phases according to card numbers 86–1629, 15–0776, and 89-2910, respectively. Figure 6 displays the XRD of FeAl20Si20 alloy and its hybrid nanocomposites with different fly ash and VC content after 10 h of mechanical milling. For Fe alloy, the intermetallic phases appear to be composed of two types of iron silicides (FeSi and Fe_3_Si) and the Fe_3_Al_2_Si_3_ ternary phase, as well as iron aluminates (FeAl), according to standard cards 65-3005, 87-1920, and 65-3201, respectively. The XRD pattern belonging to the FeSi intermetallic phase appears at 2θ = 28.58°, 45.94°, and 50.61°, the Fe_3_Si phase appears at 2θ = 45.32°, 66.03°, and 83.27°, while FeAl and Fe_3_Al_2_Si_3_ phases appear at 2θ = 43.99° and 45.55°, respectively. Based on the previous standard cards, the intermetallic phases FeSi, Fe_3_Si, and FeAl show a cubic crystal structure, while the Fe_3_Al_2_Si_3_ phase shows an anorthic crystal structure. Also, for the intermetallic alloy containing various hybrid reinforcements, the VC and fly ash hybrid do not appear except in samples FV4 and FV8, in which VC appears at 2θ = 60.89°, 36.15°, and 41.99°. In contrast, the SiO_2_ phase appears at 2θ = 26.62° and mullite phase appears at 2θ = 26.27°, 35.28°, and 40.87°. Notably, the characteristic peaks of VC, SiO_2,_ and mullite phase do not appear in the XRD chart of samples FV1 and FV2 due to their presence in tiny amounts that lie under the XRD device’s detection limit. It is also seen in the rising volume percent of the hybrid VC and fly ash to intermetallic alloy; the width of peaks increases, and the intensity decreases^40^. Accordingly, the crystal size, lattice strain, and dislocation density values of all samples are listed in Table 4. This table shows that the crystal size decreases to 19.86 nm, and the lattice strain and the dislocation density rise to 0.2467 and 2.54 × 10^−3^%, respectively, for the FV8 sample.

**Table 4:** Crystal size, lattice strain and dislocation density of various sintered sample.

Sample code	Crystal size (nm)	Lattice strain (%)	Dislocation density (%)
FV0	32.75	0.2859	9.32 × 10^−4^
FV1	30.06	0.2699	11.07 × 10^−4^
FV2	28.12	0.2042	12.65 × 10^−4^
FV4	24.12	0.2030	17.18 × 10^−4^
FV8	19.86	0.2467	25.35 × 10^−4^

### Characteristics of sintered nanocomposites

#### Physical properties

**Fig. 7:**
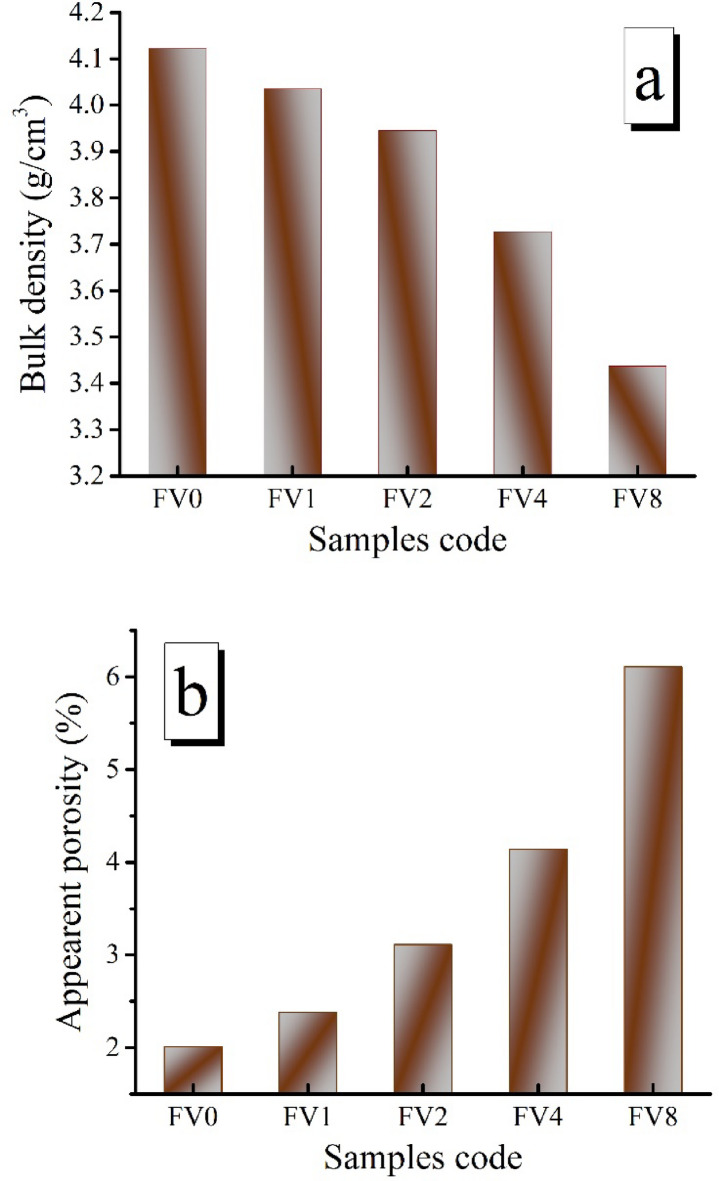
**a**) Bulk density and **b**) apparent porosity of all samples sintered at 1100 ℃ for one hour an argon atmosphere.

Figure 7(a, b) shows the effect of various volume percentages of ceramic reinforcements on the apparent porosity and bulk density of intermetallic alloy matrices sintered for one hour at 1100 °C in an argon atmosphere. As reinforcement percentages are increased, it can be seen that the intermetallic alloy matrix’s apparent porosity increases while the bulk density of the matrix reduces. The bulk density values of sintered FV0 and FV8 samples are 4.12 and 3.44 g/cm^3^, respectively. For the same samples, the apparent porosity is 2.01% and 6.11%, respectively. This may be due to increasing hybrid ceramics contents in the intermetallic base, which reduces the nanocomposite’s compacting capacity due to the higher hardness of the VC and fly ash. Also, the added hybrid reinforcements directly affect the contacts between the intermetallic phases of the matrix, grain growth, and formation of closed pores^41,42^. Finally, the addition of ceramic harms the sintering process, as the melting point of VC (~ 2810 °C) and fly ash (~ 1710 °C) is much larger than the Fe (~ 1538 °C), Al (~ 660 °C), and Si (~ 1400 °C) result in reduced the rearrangement of particles of intermetallic alloy based during sintering.

#### Microstructure of the sintered samples

**Fig. 8:**
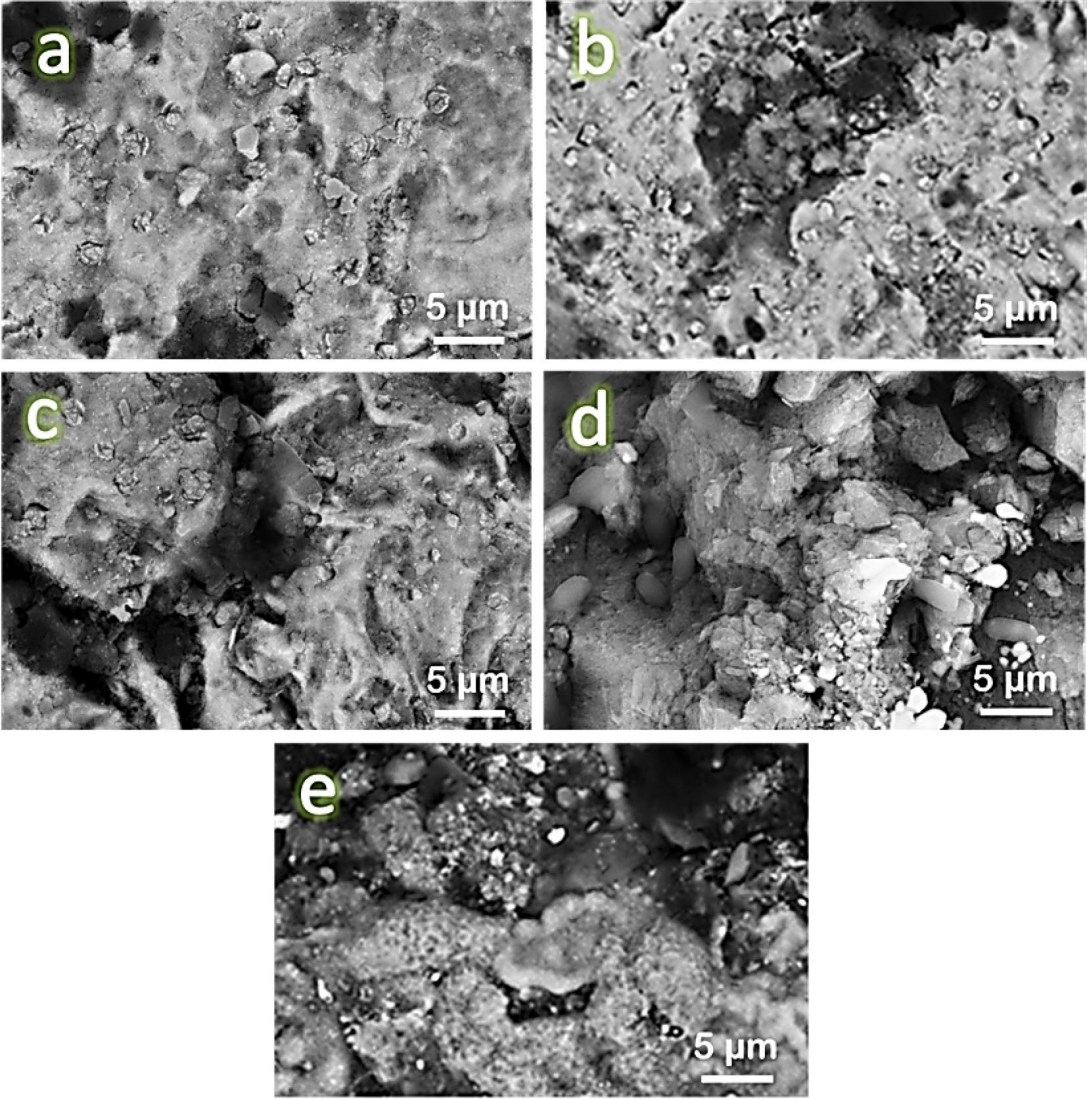
FESEM images of **a**) FV0, **b**) FV1, **c**) FV2, **d**) FV4, and **e**) FV8 samples sintered at 1100 ℃ for one hour an argon atmosphere.

**Fig. 9:**
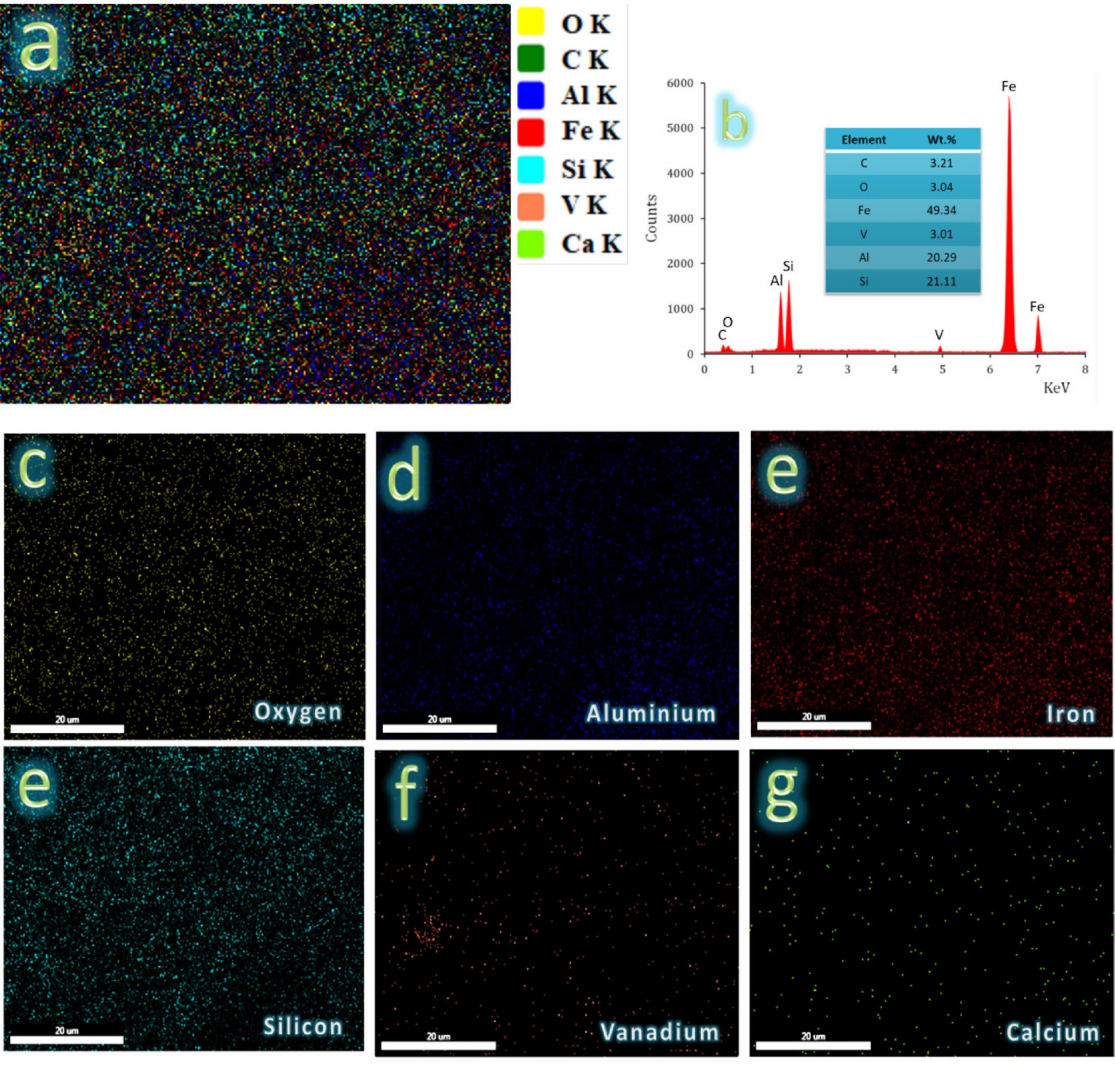
(**a**) EDX mapping of all constituents of FV8 sample, (**b**) EDX spectra of FV8 sample, and elemental mapping of the constituents forming FV8 sample, i.e., (**c**) oxygen, (**d**) carbon, **e**) aluminum, (**f**) iron, (**g**) silicon, (**h**) vanadium, and **i**) calcium.

Figure 8(a-e) displays SEM photos of the sintered FV0, FV1, FV2, FV4, and FV8 samples, respectively. Generally, selecting a suitable sintering temperature of 1100 °C encourages the diffusion process throughout the heating phase, increases densification behavior, and almost reaches excellent density. The intermetallic base (FV0) exhibited acceptable densification, as evidenced by the significant particle growth consistent with a limited number of pores. For nanocomposite samples, it is known that ceramic particles are found along the grain boundaries of the intermetallic alloy matrix. As can be seen, there is a good distribution of ceramic particles within the intermetallic alloy matrix because VC and fly ash reinforcements and intermetallic base particles were mixed well and at the right moment, which improved the wettability between them. This good distribution of reinforcements in the intermetallic base is necessary to improve the required characteristics of the nanocomposite, such as its mechanical qualities, wear resistance, and CTE value. Increasing the ceramics particles may also cause a reduced grain size of particles after milling, which may decrease the amount of agglomerated region that remains after sintering. Furthermore, it was discovered that the quantity of hybrid reinforcement particles in the samples influenced the porosity of the examined nanocomposite samples. Figure 9 indicates that EDS with elemental mapping distribution was used to analyze the hybrid nanocomposite containing a high ceramic volume percent (FV8). Figure 9a shows the homogeneous distribution of the particles composing sample FV8, which consists of carbon 3.12 wt%, oxygen 3.04 wt%, iron 49.43 wt%, vanadium 3.01 wt%, aluminum 20.29 wt%, and silicon 20.21 wt%. (Fig. 9b). Figure 9c and g show the distribution of each element separately, and it is clear that the distribution is homogeneous.

#### Thermal expansion

**Fig. 10:**
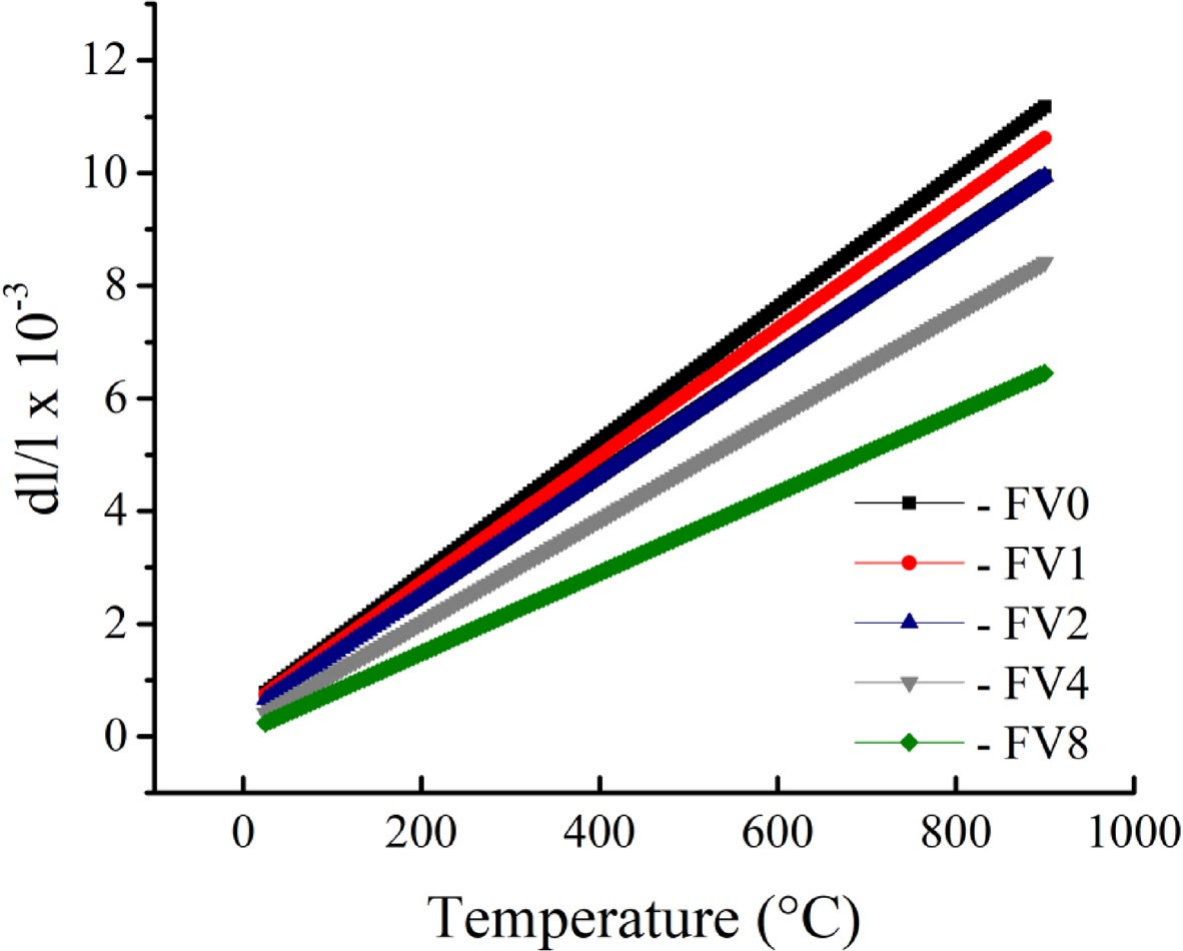
dl/l for all examined samples measured at temperatures ranging from 30 to 900.

**Fig. 11:**
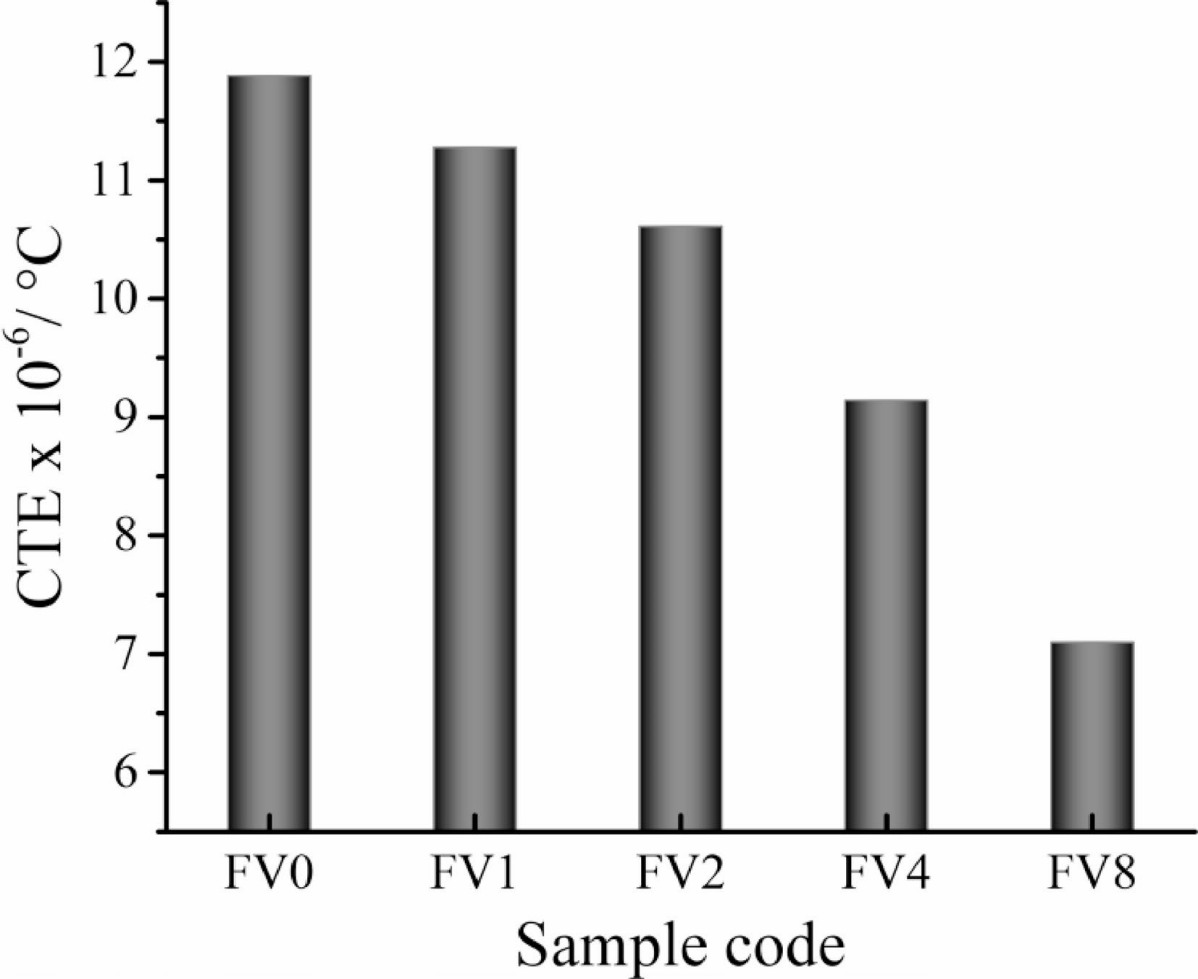
CTE values for all examined samples were measured at temperatures ranging from 30 to 900.

Figures 10 and 11 show the effect of incorporating various hybrid VC and fly ash reinforcements on the relative thermal expansion (dL/L) and the CTE value of the intermetallic alloy matrix. The findings demonstrate that the increased temperature positively impacts the dL/L value, while adding hybrid ceramics has a negative effect. As an illustration, the values of dL/L recorded at 100 °C are 1.68 × 10^−3^, 1.60 × 10^−3^, 1.46 × 10^−3^, 1.10 × 10^−3^, and 0.78 × 10^−3^. Furthermore, raising the measurement temperature to 900 °C, the values of dL/L for the identical samples are 11.17 × 10^−3^, 10.62 × 10^−3^, 9.94 × 10^−3^, 8.41 × 10^−3,^ and 6.45 × 10^−3^. It is evident that as the volume fraction of the ceramics in the intermetallic alloy matrix increases, the value of CTE decreases (in the same direction as the dl/l value). The CTE values of FV0 sample (intermetallic alloy) is 11.88 × 10^−6^/⁰C, while for FV1, FV2, FV4 and FV8 nanocmposites samples the values are 11.28 × 10^−6^/⁰C, 10.61 × 10^−6^/⁰C, 9.14 × 10^−6^/⁰C and 7.10 × 10^−6^/⁰C, respectively. This decrease in the values ​​of dl/l and CTE of nanocomposite samples compared to unreinforced intermetallic alloy is due to the presence of vanadium and silica, which have a low CTE (~ 7.2 and > 1 × 10^−6^/⁰C, respectively) value compared to the matrix (12.8 × 10^−6^/⁰C). Moreover, the value of CTE strongly correlates with the density of samples, as increasing the density leads to an increase in the value of CTE due to the development of microstructure and densification manner. Accordingly, the sample density decreases with increasing stripping and thus leads to a decrease in the CTE value of the samples^43,44^.

Thermal stability is significantly increased when hybrid VC and fly ash are added to intermetallic alloys, especially under varying industrial operating conditions such as industrial settings where oxidation, thermal cycling, and variable temperatures are common. This is a quick but thorough look:

- Fly ash and VC ceramics are examples of thermally stable reinforcements that slow down diffusion processes and inhibit grain formation. This enables the microstructure to be preserved even when subjected to increased temperatures.
- Due to the high melting point and low thermal conductivity of ceramics, reinforced intermetallic alloys with hybrid VC and fly ash maintain rigidity and resist softening over time. In contrast, intermetallic alloys alone generally soften or oxidize in high-temperature applications (600–1100 °C) or heat exchangers.
- By reducing the mismatch in CTE value caused by hybridization, hybrid ceramics increase resistance to thermal fatigue and creep. Additionally, stress redistribution at the ceramic-matrix contacts causes delayed fracture propagation.
- As previously mentioned, the addition of hybrid ceramics has a positive effect on improving the coefficient of thermal expansion (CTE) value, as the addition of hybrid ceramics increases their resistance to thermal fatigue and creep. Additionally, stress redistribution at the ceramic-matrix contacts causes delayed fracture propagation.
- Corrosion and oxidation resistance, in which ceramics function as diffusion barriers or create stable oxide layers.

#### Mechanical properties

**Fig. 12:**
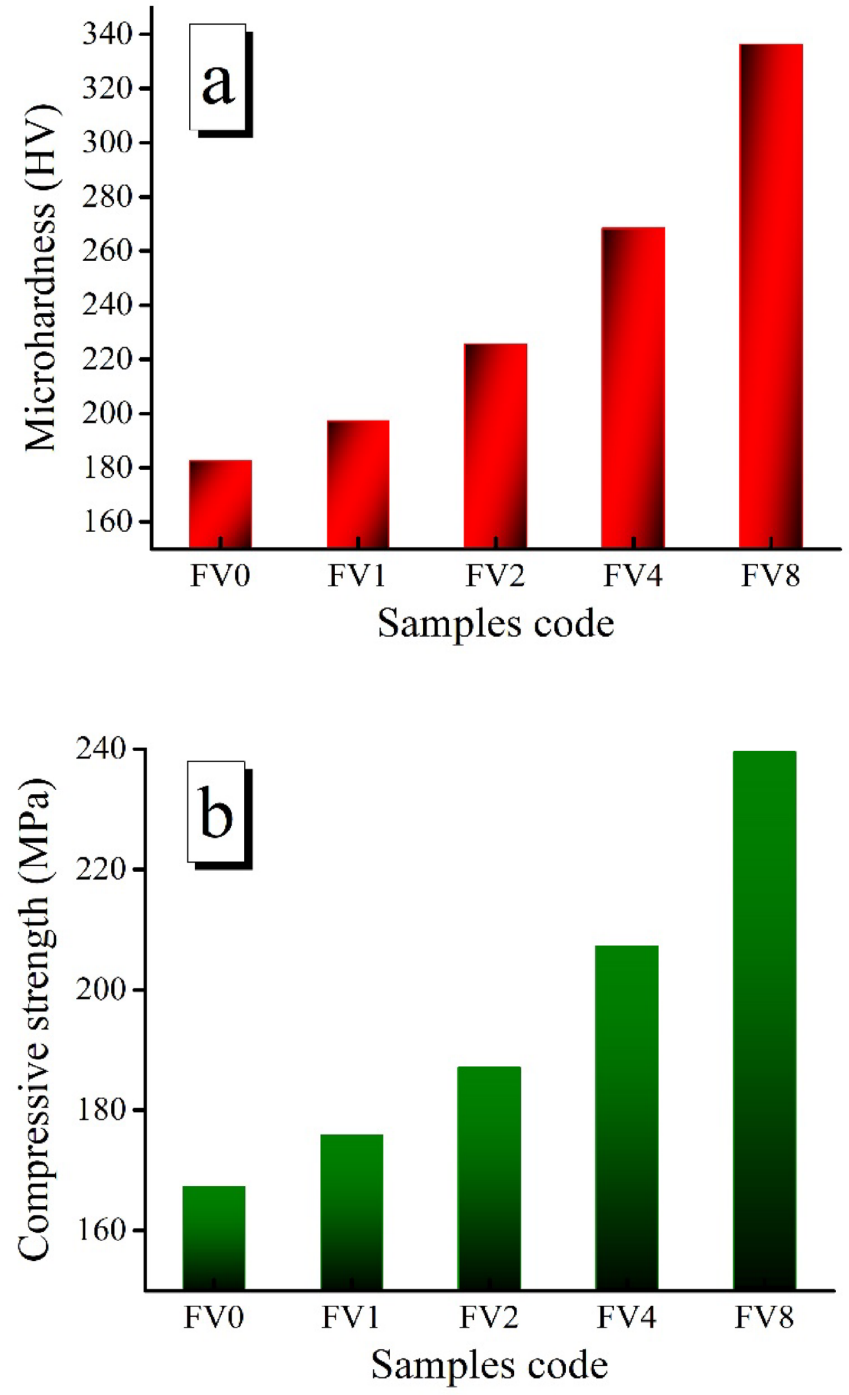
(**a**) Microhardness and (**b**) compressive strength of all examined samples.

**Fig. 13:**
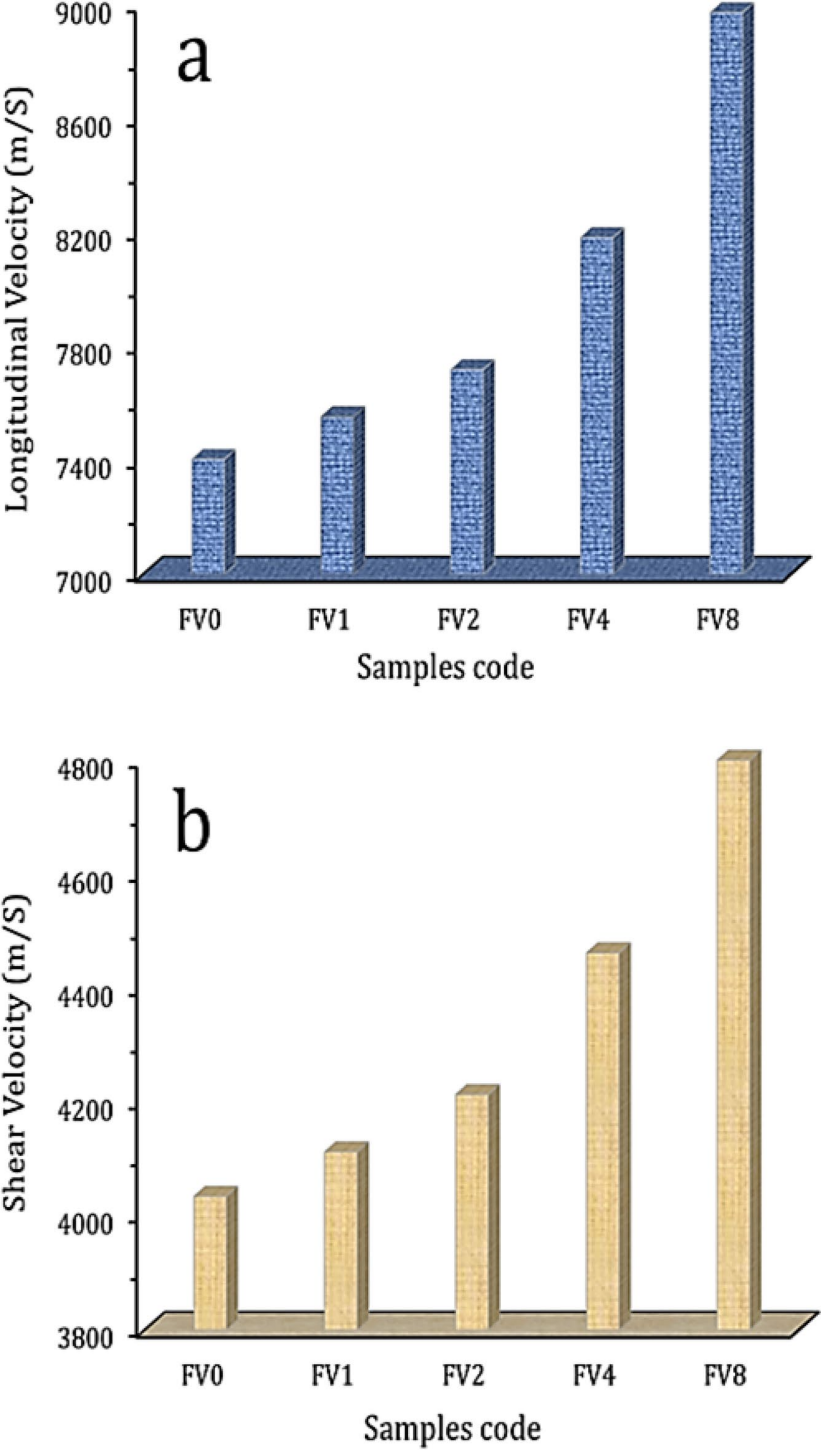
Variations in **a**) longitudinal ultrasonic velocity and **b**) shear ultrasonic velocity of all examined samples.

**Fig. 14:**
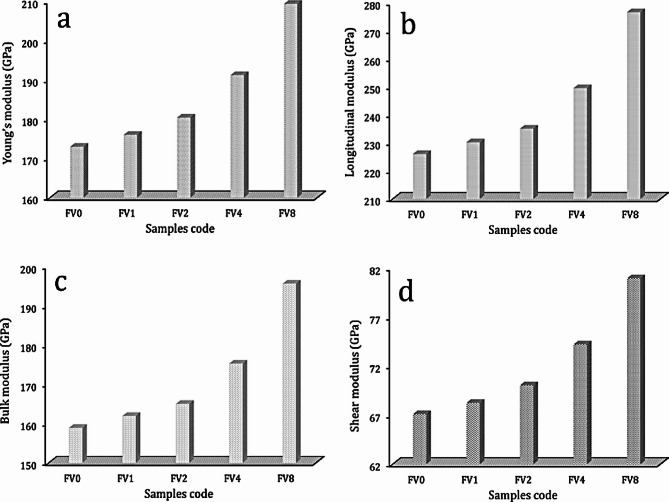
Variations **a**) Young’s modulus, **b**) longitudinal modulus, **c**) bulk modulus, and **d**) shear modulus of all examined samples.

Figure 12(a, b) depicts the impact of hybrid ceramics on Vickers microhardness and compressive strength values of the intermetallic alloy matrix. According to the data shown in the figure, increasing VC and fly ash volume percent is the main factor leading to the improvement of microhardness values. Notably, microhardness values are 182.47, 197.28, 225.57, 268.42, and 336.23 Hv while compressive strength is 167.25, 175.81, 187.09, 207.23, and 239.54 MPa for intermetallic alloy matrix containing 0, 2, 4, 8, and 16 vol% hybrid reinforcement. As illustrated in Figs. 13 and 14, other mechanical properties of the nanocomposite samples were also evaluated utilizing ultrasonic velocities and the elastic moduli group. This figure clearly shows that ultrasonic velocity and all elastic moduli exhibit the same trend as microhardness, which was previously mentioned, and show considerable increases in their values as the proportion of hybrid ceramics increases. The longitudinal and the shear velocities values for the FV0 sample are 7504.32 and 4034.25 m/s, respectively, while the value of longitudinal velocity for FV1, FV2, FV4, and FV8 nanocomposite samples, the value are 7552.44, 7718.23, 882.22, and 897,128 m/s, respectively and for the identical specimens, the value of shear velocity are 4112.35, 4213.08, 4462.24, and 4853.02 m/s, respectively. Also, the values of Young’s and shear moduli of FV8 nanocomposite samples are 209.34 and 80.94 GPa, which increased by about 21% and 20.7%, respectively, compared to the FV0 specimen, which recorded 172.95 and 67.09 GPa, respectively.

The enhancement of the mechanical properties of the intermetallic alloy after the incorporation of hybrid reinforcements of VC and fly ash may be ascribed to many causes:

##### Orowan strengthening

The Orowan strengthening effect plays a vital role in enhancing the mechanical properties of intermetallic alloys, resulting from the homogeneous dispersion of hard hybrid reinforcement phases into the intermetallic alloy, which acts as a barrier for dislocation movement. As a result, dislocation loops are created around VC and fly ash particles, causing an increase in the stress required for further deformation^45–47^.

This enhancement in microhardness of intermetallic alloy after adding hybrid reinforcements can be better understood by noting the following Eq. (11)^48^.

11 $$\:\text{H}\text{v}=\:{H}_{I}{V}_{I}+\:{H}_{V}{V}_{V}+\:{H}_{F}{V}_{F}$$

where H_I_, H_V_, and H_F_ are the microhardnesses of the intermetallic alloy, VC, and fly ash, respectively, while V_I_, V_V_, and V_F_ are the volume fractions of the intermetallic alloy, VC, and fly ash, respectively.

##### Thermal-mismatch strengthening

Thermal mismatch strengthening is related to the significant difference between the CTE of the intermetallic alloy matrix and reinforcements (VC and fly ash) particles, contributing to producing thermally induced residual stresses^49,50^. Even with small temperature changes, the thermal stresses generated in the intermetallic alloy matrix significantly contribute to high dislocation density in the vicinity of the interface and, therefore, strengthen the nanonanocomposite.

##### Load transfer from the intermetallic alloy to the reinforcements

In the compressive testing, the load transfer between the hard reinforcement particles and the intermetallic alloy, especially if the connection between reinforcement particles and intermetallic alloy is good enough, as shown by Eq. (12)^51,52^:

12 $$\:{{\upsigma\:}}_{\text{l}\text{o}\text{a}\text{d}}=0.5\:{\text{V}}_{\text{f}}{{\upsigma\:}}_{\text{Y}}\:$$

where σ_load_ is load transfer, V_f_ represents the volume fraction of the hybrid reinforcements phase, and σ_Y_ represents the yield stress of the intermetallic alloy.

The use of various ceramic materials to enhance the mechanical characteristics of intermetallic alloys has been the subject of many papers. For instance, AbuShanab et al.^15^ added 10% VC and 10% fly ash to the Al-Si alloy, and they found that its microhardness increased by 75%. However, Aranda et al.^53^ added chromium (Cr) at different concentrations to the Al-Si-Fe alloy produced by two solidification methods. They found that in conventional solidification, the alloys containing five wt% Cr (220 HV) exhibited the maximum microhardness value, and the three wt% Cr showed the highest microhardness in vacuum casting at 192 HV.

### Wear analysis

**Fig. 15:**
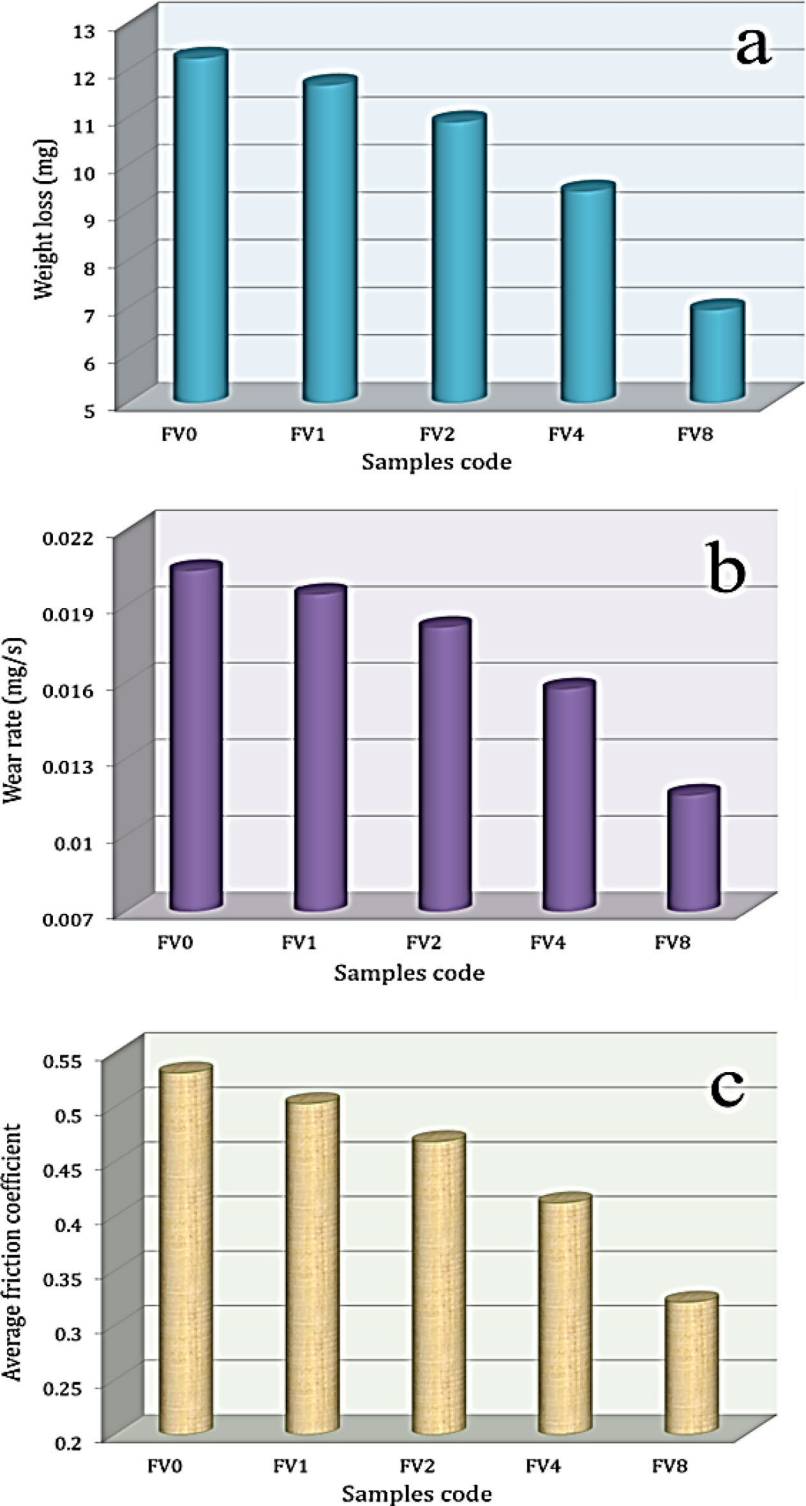
**a**) Weight loss, **b**) wear rate, and **c**) average friction coefficient of all sintered samples.

The effect of incorporating VC and fly ash on weight loss, wear rate, and average friction coefficient of sintered samples is displayed in Fig. 15(a-c). The findings indicate that samples’ weight loss, wear rate, and average friction coefficient tend to decrease with increasing hybrid reinforcement volume percent. For the nanocomposite samples FV1, FV2, FV4, and FV8, the wear rate decreases to 0.0195, 0.0182, 0.0157, and 0.0116 mg/s, which represents a reduction of about 4.6%, 10.9%, 22.8%, and 43.2%, respectively, compared to the FV0 sample (0.0204 mg/s). Moreover, the average fraction coefficient of previous nanocomposite samples is 0.503, 0.468, 0.412, and 0.3338, which is reduced by about 5.3%, 11.9%, 22.4%, and 39.5%, respectively, compared to the FV0 sample (0.531). Adding hybrid reinforcements positively impacts the produced samples, enhancing wear resistance effectively. It is crucial to highlight that, as was previously mentioned, the addition of reinforcements to the alloy base results in an enhancement in the mechanical properties of the samples^54^, which in turn causes the wear rate (w) to decrease by Archard’s Eq. (13)

13 $$\:w=\frac{KP}{H}$$

Where K is the constant value, P is the load, and H is the microhardness of the nanocomposite sample. Another reason for the decrease in wear rate and fraction coefficient behavior of hybrid ceramics is that they can tolerate the contact load between the two surfaces, which results in soft intermetallic compound alloy protection from wear^55,56^. Several researchers have studied the effect of various ceramic materials on enhancing the tribological properties of Al-based alloys. For instance, Walczak et al.^57^. conducted experiments on an AlSi12CuNiMg casting alloy supplemented with fine graphite particles at 5.7 wt%. They discovered that the friction coefficient had dropped by 8.9%. Moustafa et al.^58^. created Al-4.2-Cu-1.6Mg matrix nanocomposites reinforced with nano-ZrO_2_ particles utilizing powder metallurgical techniques. The findings revealed that adding 16 wt% ZrO_2_ reduced the wear rate by about 37.5%.

## Conclusions

A mixture of different powder batches combined using powder metallurgy with subsequent conditions led to the formation of an intermetallic compound and its composites with an ultra-fine microstructure composed of FeSi, Fe_3_Si, FeAl, and Fe_3_Al_2_Si_3_ phases as matrix and SiO_2_, mullite, and VC as reinforcements. The following are the conclusions:

- After the milling process, it should be noted that adding various percentages of VC and fly ash particles increased the grain refining of the intermetallic compound alloy and reduced the crystallite size of the composite powders to ultrafine ranges of about 17 nm.
- The bulk density of the intermetallic base gradually reduced with an increased amount of ceramics from 4.12 g/cm^3^ to 3.44 g/cm^3^, while porosity rose from 2.01 to 6.11%.
- The increased temperature positively impacts the dL/L value, while adding hybrid ceramics has an adverse effect. The recorded values of dL/L at 100 °C are 1.68 × 10^−3^, 1.60 × 10^−3^, 1.46 × 10^−3^, 1.10 × 10^−3^, and 0.78 × 10^−3^. Furthermore, raising the measurement temperature to 900 °C, the values of dL/L for the identical samples are 11.17 × 10^−3^, 10.62 × 10^−3^, 9.94 × 10^−3^, 8.41 × 10^−3^, and 6.45 × 10^−3^.
- The CTE values of the prepared samples decreased from 11.88 × 10^−6^/⁰C to 7.10 × 10^−6^/⁰C, reflecting the positive effect of ceramic materials on improving the thermal stability of the prepared samples.
- Gradual improvement of the mechanical properties of the intermetallic compound alloy, including microhardness, hardness, and modulus of elasticity, after adding different percentages of hybrid ceramic. For example, the microhardness values of FV0, FV1, FV2, FV4, and FV8 are 182.5, 197.3, 225.6, 268.4, and 336.2 Hv, respectively, and the longitudinal modulus of the same previous samples is 225.9, 230.2, 235, 249.5, and 276.6 GPa, respectively.
- The wear resistance of the intermetallic base improved after adding hybrid ceramics, and the wear rate and coefficient fraction values decreased dramatically with the increase in ceramics content. The coefficient fraction of the intermetallic compound alloy is 0.531, and after adding 2, 4, 8, and 16 vol% hybrid ceramic, the value of the coefficient fraction decreases to 0.503, 0.468, 0.412, and 0.338, respectively.
- Given the encouraging findings, expanding these investigations to an industrial level is feasible by modifying the powder metallurgy method for high-throughput systems, including industrial mixing, pressing, and sintering apparatus.

## Data Availability

The datasets generated and/or analyzed during the current study are not publicly available because all data are presented in the article. Therefore, there is no need to include raw data, but they are available from the corresponding author upon reasonable request.
